# Ruptured Renal Abscess From Streptococcus agalactiae Invasion in a Postpartum Female

**DOI:** 10.7759/cureus.15701

**Published:** 2021-06-16

**Authors:** Keith B Wright, Kathryn M Burtson

**Affiliations:** 1 Internal Medicine, Wright-Patterson Air Force Base/Wright State University, Dayton, USA; 2 Internal Medicine, Wright State University Boonshoft School of Medicine, Dayton, USA

**Keywords:** postpartum fever, non-cardiac chest pain, group b streptococcus (gbs), perirenal abscess, pararenal abscess, bacterial abscess, renal abscess, vaccine science and policy, vaccine acceptance

## Abstract

*Streptococcus agalactiae *(Group B *Streptococcus* or GBS)is an exceptionally rare causative organism of a ruptured renal abscess. We report a case of this normally commensal organism causing a large ruptured renal abscess in a 17-year-old postpartum female. Although *S. agalactiae* is known to cause postpartum neonatal morbidity and mortality, it has rarely caused invasive infections in the last 20 years in adults. While this diagnosis often presents with nonspecific findings that can easily be overlooked during the postpartum period, the patient responded well to the established treatment of broad-spectrum antibiotics and a percutaneous drain.

## Introduction

Perinephric and intranephric abscesses are well-documented infections that typically have a nonspecific presentation and are often diagnosed on opportunistic or incidental imaging [1]. These abscesses usually develop from an ascending urologic infection of gram-negative bacilli, with the most common isolates being *Escherichia coli* and *Klebsiella pneumoniae* [2,3]. The infectious organism can spread beyond the capsule of the kidney leading to a secondary infection and even invade other surrounding structures either by local migration or rupture of the renal capsule. Renal capsule rupture is uncommon, occurring in only 10% of intranephric abscesses, and is most often caused by gram-positive organisms [4]. *Staphylococcus aureus* is the most common isolate with a few rare cases involving other organisms, including group B *Streptococcus* (GBS) [5,6]. In published cases of confirmed GBS intranephric or combined perinephric abscesses, the hosts were immunosuppressed, with diabetes being the most common comorbidity [7]. During pregnancy and the puerperium, several physiologic changes contribute to urinary stasis and vesicoureteral reflux which increase the susceptibility to urinary tract infections. These physiologic changes include increased bladder volume, decreased bladder and ureteral tone, compression of the bladder and ureters by the gravid uterus, and increased urinary progestins and estrogens [8]. While urinary tract infections and progression to pyelonephritis are common in pregnancy due to urinary tract alterations, the development of a renal abscess either during pregnancy or following delivery is extremely rare [9].

## Case presentation

A generally healthy 17-year-old female G1P1001 presented to a routine two-week postpartum visit complaining of chest pain. Of note, her pregnancy was detected incidentally in her second trimester when she was treated for *E. coli* pyelonephritis. At 35 weeks, she was found to be positive for GBS and received two doses of ampicillin prior to delivery. The spontaneous vaginal delivery was complicated by chorioamnionitis, which was treated with ampicillin and gentamicin. On the resolution of chorioamnionitis, the patient was discharged with a plan to complete four weeks of nitrofurantoin.

**Figure 1:**
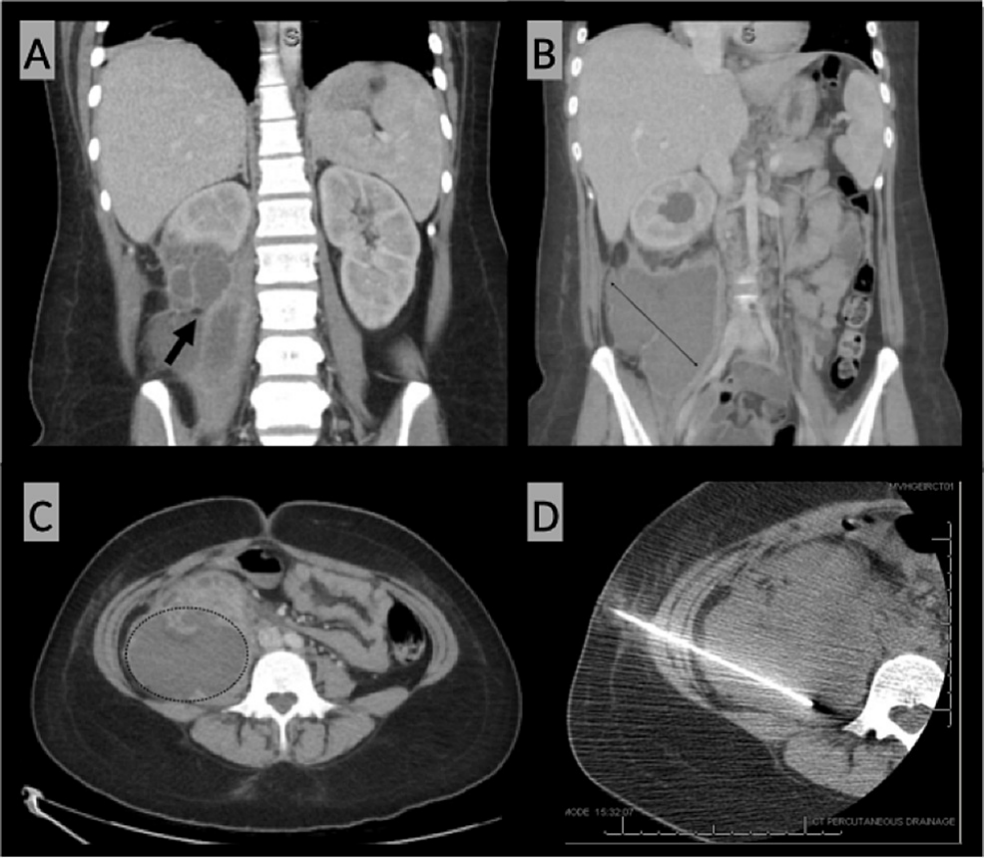
(A) Abdomen/pelvis CT (coronal view) illustrates the defect in the right renal capsule (arrow) indicating the rupture of the intranephric abscess into the perinephric space. (B) Abdomen/pelvis CT (coronal view) demonstrates the coronal dimensions of the abscess. (C) Abdomen/pelvis CT (transverse view) demonstrates the transverse dimensions of the abscess. (D) CT imaging during interventional radiology procedure showing successful drain placement. CT: computed tomography

At her two-week postpartum visit, she presented with pleuritic chest pain in the middle of her chest and below her ribs bilaterally. The pain was exacerbated when lying on the back or on the right side. The patient was sent home with standard return instructions. One week later, she presented to the emergency department for persistent chest pain with radiation to her right abdomen, as well as fever, nausea, and vomiting. At this time, the patient was febrile with a temperature of 100.6°F, had an elevated heart rate of 105 beats/minute, and was normotensive. On examination, both upper abdominal quadrants were tender to palpation but there was no costovertebral angle (CVA) tenderness. A computed tomography (CT) scan of her abdomen and pelvis revealed a 10 × 10 × 18 cm intranephric abscess with renal capsule rupture and invasion of the perinephric space (Figure 1A-1C). The patient was started on piperacillin-tazobactam prior to emergent percutaneous drain placement (Figure 1D). Cultures of the drained fluid were positive for pansensitive GBS. The patient responded well to therapy and was discharged home to complete a three-week course of amoxicillin.

## Discussion

Perinephric and intranephric abscesses occur in an estimated 10 cases per 10,000 admissions [1]. Rupture of the renal capsule is associated with further complications leading to higher mortality. Potential complications include sepsis, bleeding, fistula formation (to the stomach, small bowel, or lung), subphrenic abscess, rupture into the peritoneum, perforation of the diaphragm, and empyema. Although the most common presenting symptoms are nonspecific, they commonly include fever or chills, flank pain, abdominal pain, nausea and vomiting, anorexia, and/or fatigue. CVA tenderness on the ipsilateral side is commonly present [6]. Given the frequently nonspecific presentation, the diagnosis is generally made on imaging, with CT being the preferred modality.

GBS is a common commensal bacterium with colonization rates estimated at 22% in adults. In a recent large cohort study, only 0.1% of postpartum females were found to develop invasive GBS disease [10]. In the general population, only a few cases have been reported with GBS as the causative organism of intranephric or combined perinephric abscesses. In all of these reported cases, a predisposing condition was found, most commonly diabetes. In the past few decades, the prevalence of invasive GBS infections has more than doubled. The reason for this increase in prevalence is not yet clear but is associated with the increasing prevalence of predisposing chronic conditions and the increasing mean age of the global population [7,11,12]. Predisposing conditions include diabetes mellitus, pregnancy, liver cirrhosis, chronic renal insufficiency, malignancy, previous urological instrumentation, and structural abnormalities of the urinary tract.

The mortality rates of intranephric and perinephric abscesses range from 12% to 56%, with higher mortality attributed to delay in diagnosis [13-15]. Management of perinephric abscesses includes antibiotic therapy with concurrent drainage for abscesses larger than 3 cm. Abscesses smaller than 3 cm may be treated with antibiotics alone. The duration of antibiotics should extend from two to three weeks with close follow-up for persistent clinical symptoms and further drain management [3].

While a rare cause of renal abscesses, primary prevention of invasive GBS infections may be on the horizon. After a promising phase I/II clinical trial, GBS conjugate vaccination in high-risk adults may be possible in the near future [16,17]. Given the increasing incidence of GBS invasive infections, this case presents a timely narrative of the potential benefit of a GBS vaccine for females of child-bearing age. The prevalence of vaginal or rectal colonization in gravid females ranges between 10% and 30%. Currently, preventative strategies are in place with prenatal GBS screening and intrapartum prophylactic antibiotic treatment as indicated. These measures are targeted at reducing the rates of vertical transmission to the neonate. As an additional measure, preconception GBS vaccination has the potential to mitigate the risks of both maternal and neonatal morbidity and mortality in the setting of GBS-positive pregnant women [18].

## Conclusions

Although invasive GBS infections are increasing in incidence, they remain a rare cause of renal abscesses. Presentation is frequently nonspecific, with diagnosis made on CT imaging. Delay in diagnosis and rupture of the renal capsule result in higher mortality rates. In the postpartum state, diagnosis can be delayed. The current standard of care focuses on preventative strategies to reduce the morbidity and mortality of the neonate, with routine prepartum screening and appropriate administration of intrapartum antibiotics. A GBS conjugate vaccine is under trial for high-risk adults, which has the potential of becoming an important component of patient-provider discussions regarding both preconception and postconception planning. Cases such as this can be prevented with the advent of an effective vaccine against GBS.
